# Effect of Melatonin on Increasing the Effectiveness of Liver Preservation Solution

**DOI:** 10.5152/tjg.2023.22694

**Published:** 2023-09-01

**Authors:** Mehmet Mustafa Erdoğan, Mehmet Erman Erdemli, Onural Özhan, Zeynep Erdemli, Harika Gözde Gözükara Bağ, Nigar Vardı

**Affiliations:** 1 Department of Histology and Embryology, Malatya Education and Research Hospital, Malatya, Turkey; 2 Department of Medical Biochemistry, İnönü University Faculty of Medicine, Malatya, Turkey; 3 Department of Medical Pharmacology, İnönü University Faculty of Medicine, Malatya, Turkey; 4 Department of Biostatistics and Medical Informatics, İnönü University Faculty of Medicine, Malatya, Turkey; 5 Department of Histology and Embryology, İnönü University Faculty of Medicine, Malatya, Turkey

**Keywords:** Liver, melatonin, preservation, UW solution

## Abstract

**Background/Aims:**

Various tissue preservation solutions are used during the removal of the organ and during transplantation to protect the normal histological and biochemical characteristics of tissue while performing a successful liver transplant. In our study, it was aimed to investigate the effects of intraperitoneal melatonin administration on liver preservation damage before transplantation.

**Materials and Methods:**

In our study, the histological and biochemical characteristics of University of Wisconsin + melatonin group rats treated with melatonin 45 minutes before hepatectomy were compared between serum physiologic group and University of Wisconsin group.

**Results:**

When hematoxylin and eosin staining was evaluated in terms of hydropic degeneration, sinusoidal dilatation, and hepatocyte necrosis, there was no statistically significant difference. Caspase 3 immunohistochemical staining showed a significant increase in Caspase 3 immunoreactivity positivity at the 12^th^-hour University of Wisconsin group compared to University of Wisconsin + melatonin group. As a result of biochemical analysis, the malondialdehyde and total oxidant status levels in the University of Wisconsin + melatonin group decreased significantly compared to the University of Wisconsin group. When the reduced glutathione activity and total antioxidant capacity level were compared, a significant increase was observed in the University of Wisconsin + melatonin group compared to the University of Wisconsin group at the 12^th^ hour. It was also found that aspartate aminotransferase, alanine aminotransferase, and alkaline phosphatase levels decreased significantly in the University of Wisconsin + melatonin 12^th^-hour group compared to the University of Wisconsin 12^th^ hour and control group.

**Conclusion:**

When the findings were evaluated, intraperitoneal administration of melatonin, a cytoprotective antioxidant, was found to play an effective role in preserving immunohistochemical and biochemical properties of liver tissue integrity and hepatocytes in University of Wisconsin solution.

**Main Points**

- According to the histological findings, a significant decrease was observed in the melatonin group in terms of necrosis, hydropic degeneration, and sinusoidal dilatation in liver cells.
- When Caspase 3 was evaluated in terms of immunoreactivity, intense Caspase 3+ was observed in the University of Wisconsin (UW) group, while a significant decrease was found in the melatonin group.
- In the biochemical analyses, it was determined that the malondialdehyde and total oxidant status levels increased in the UW group and decreased significantly in the melatonin group. In addition, reduced glutathione and total antioxidant capacity (TAC) levels decreased in the UW group but increased in the melatonin group. Aspartate aminotransferase, alanine aminotransferase, and alkaline phosphatase values of liver enzymes were found to be significantly lower in the melatonin group compared to the control and UW groups.
- Intraperitoneal administration of melatonin before organ transplantation played an important role in terms of cellular and biochemical preservation of liver tissue in UW solution.

## Introduction

The main purpose of organ preservation is to preserve the cellular and biochemical structure of the organ taken from the donor at the highest possible level until it is transplanted and to continue its functional activity after transplantation.^1^

In the last quarter century, 160-170 different preservation solutions have been defined. However, histidine-tryptophan-ketoglutarate (HTK) and University of Wisconsin (UW) solutions have come to the fore among the preservation fluids that are being widely used and studied all over the world.^1–3^ Preventing cellular swelling, removing the blood inside the organ, minimizing free radical-induced organ damage, and providing hypothermia are important in the basis of organ preservation techniques.^1,4^ Liver taken from a cadaver for transplantation is primarily preserved in UW solution under hypothermic conditions during the transfer process.^5^

The UW solution developed by Belzer and Southard provides extended preservation periods for kidney, liver, and pancreas in clinical and experimental transplantation.^6^ This solution made organ sharing possible even over long distances, making liver transplantation a more elective procedure rather than an emergency procedure. Safe preservation is provided for the liver for up to 24 hours in UW solution under suitable transferring conditions.^5^ Despite improvements in preservation, the rate of graft dysfunction still varies between 5% and 20%.^7^

Melatonin is a hormone that plays a role in the regulation of many biological functions.^8,9^ Following its synthesis, it is delivered directly to the circulation through the pineal gland. Cytosolic and nuclear binding sites have also been defined for melatonin, which can enter all compartments of cells thanks to its lipophilic feature.^10^ Thus, melatonin can effectively protect cell membranes, organelles, and nuclei from free radical damage. In the presence of melatonin, the production of radicals from the mitochondrial respiratory chain is also reduced. The ability of melatonin to reach the nucleus provides superiority to it in protecting deoxyribonucleic acid against oxidative damage.^11,12^ Various studies indicate that melatonin is effective in preventing oxidative liver damage.^13^

In our study, we aimed to investigate whether or not the systemic application of melatonin, before the removal of the cadaver liver, contributes to the protection of hepatocyte and biliary system epithelial cells of the UW solution used in the liver preservation process.

## Materials and Methods

The approval was obtained from İnönü University Faculty of Medicine, Experimental Animals Ethics Committee on September 4, 2018, with the protocol number 2018/A-29. In the study, 28 Wistar Albino rats that were 10-12 months old, with an average weight of 250-300 g, were used. This experimental study was conducted on 3 groups, each consisting of randomly selected rats. The control group had 8 randomly selected rats and the other 2 groups had 10 randomly selected rats each.

Group 1 (n = 8): serum physiologic (SP, Control group)

Group 2 (n = 10): UW

Group 3 (n = 10): UW + melatonin (UW + Mel (10 mg/kg, intraperitoneal))

All the animals were anesthetized by intraperitoneal administration of 50 mg/kg ketamine hydrochloride (Ketalar® Eczacıbaşı Warner-Lambert pharmaceutical industry, Levent, İstanbul, Turkey) and 10 mg/kg xylazine hydrochloride (Rompun® Bayer, Şişli, İstanbul, Turkey) under aseptic conditions. Liver samples of rats in the SP group were kept in falcon tubes containing 10% formaldehyde. Liver samples of rats in the UW group were kept in UW solution on ice in falcon tubes. Forty-five minutes before hepatectomy was performed, the rats in the UW + Mel group were given a single dose of 10 mg/kg melatonin dissolved in 0.5 mL 5% ethanol intraperitoneally. After hepatectomy, liver samples of rats were also kept in UW solution on ice in falcon tubes. The incision part of the anesthetized rats was shaved. After the skin antisepsis was provided with povidone-iodine, the arms and legs were fixed to the surgical apparatus. The device was kept at a 30-degree inclination in order to prevent the risk of aspiration, and the sterile cover was closed in a way that the incision area was left open. A midline incision was preferred. After laparotomy, the small intestines were taken out of the abdomen, and the portal pedicle was defined.

After laparotomy was performed on rats, the portal vein was cannulated and the distal of the portal pedicle was tied. Then, the liver was perfused through the portal vein at +4°C until clear fluid came from the hepatic vein, and a hepatectomy was performed. The livers of the rats in all experimental groups were totally removed under ketamine/xylazine anesthesia and the rats were euthanized.

Liver samples were placed in falcon tubes containing 20 mL UW solution after hepatectomy and the tubes were placed in ice-filled storage containers. Samples were taken from liver samples in all groups for histological examinations at the 0 and 12^th^ hours. Liver tissue samples of 6 randomly selected rats were taken from each group for biochemical analysis at 0 and 12^th^ hours for malondialdehyde (MDA), reduced glutathione (GSH), total antioxidant capacity (TAC), and total oxidant status (TOS) analyses. The UW solution samples were taken from 6 falcon tubes randomly for aspartate aminotransferase (AST), alanine aminotransferase (ALT), and alkaline phosphatase (ALP) analysis of UW and UW + Mel groups at the 12^th^ hour. Samples taken for histological examination were placed in a 10% formaldehyde solution. Tissue and UW solution samples taken for biochemical analysis were kept at −80°C.

### Light Microscopic Techniques

The liver tissue samples of the rats taken during the experiment were fixed in 10% formaldehyde for 48 hours in a separate storage container spared for each rat. At the end of the detection, the tissues washed in tap water were buried in paraffin blocks by dehydration in the ethanol series (50%-99.9%) and polishing in the xylene series. Five-micrometer-thick sections were taken on slides from paraffin blocks with the help of a microtome (Leica RM 2145). The sections were kept in the oven at 56°C for 1 hour. The sections undergoing deparaffinization and rehydration processes were stained histochemically using hematoxylin and eosin (HE), and periodic acid Schiff (PAS) techniques. Immunohistochemical (IHC) Caspase 3 labeling was performed using the Streptavidin biotin method.

The liver tissues in the SP group were fixed in 10% formaldehyde directly. Therefore, the SP group was taken as SP 0 hour only in light microscopic evaluation and comparison between groups, since there would be no difference in terms of histological findings between hours.

The HE and PAS stained sections were obtained using entellan; IHC applied sections were covered with a coverslip using a water-based sealer. Sections were examined with Nikon Eclipse Ni-U light microscope, Nikon DS-Fi2 camera and Nikon NIS-Elements Documentation image analysis program (Nikon Corporation, Tokyo, Japan) and were photographed. In the liver sections stained with HE, 10 areas were examined at 20× objective magnification. Histopathological changes (sinusoidal dilatation, hydropic degeneration, hepatocyte necrosis) were scored from 0 to 3 (0; absent; 1, mild if in ≤25% of the liver; 2, moderately diffused if in 25%-50% of the liver; 3, diffused if in ≥50% of the liver) with a maximum total score of 9. In sections stained with PAS, intracytoplasmic glycogen staining intensity in hepatocytes was scored between 0 and 3 (0, no positive staining; 1, weak staining; 2, moderate staining; 3, strong staining). Only the intensity of immunostaining was evaluated in the sections which had IHC Caspase 3 staining. The positivity of Caspase 3 immunoreactivity was scored between 0 and 3 (0, no staining; 1, weak staining; 2, moderate intensity staining; 3, strong staining).

### Biochemical Analysis

At the end of the study, MDA, TAC, TOS levels, and GSH activity were measured in liver tissues placed into the liquid nitrogen tank and then kept in a −80°C freezer. The liver tissues kept in the deep freezer were weighed on the working day after being taken out. Phosphate buffer was added to form 10% homogenate and was homogenized in ice for 1-2 minutes at 12,000 rpm (IKA, Germany). Tissue homogenates were centrifuged at 5000 rpm, +4°C and for 30 minutes, and the supernatant was obtained.

The GSH analysis was performed according to the method described by Ellman.^14^ The MDA analysis was performed according to the method reported by Mihara and Uchiyama.^15^ In the measurement of TAC, commercial TOS measurement kit (Rel Assay Diagnostic, Turkey) was used. The principle of the method is based on the fact that the oxidants in the sample oxidize the ferrous ion-chelator complex to ferric ions and the resulting ferric ions form color with the chromogen material in an acidic environment.^16^ Commercial TAC measurement kit (Rel Assay Diagnostic) was used. The principle of the method is that the antioxidants in the sample reduce the dark blue-green 2,2’-azino-bis(3-ethylbenzothiazoline-6-sulfonic acid) (ABTS) radical to the colorless ABTS form.^17^

The UW solution samples belonging to UW and UW + Mel at the 12^th^ hour stored at −80°C until the working day were centrifuged at 1000×*g* for 10 minutes on the working day. Cloud-Clone Corp (23603 W. Fernhurst Dr, Unit 2201, Katy, TX, USA) which is a measurement method was studied with ELISA and AST, ALT, and ALP parameters were studied with brand Rat-specific ELISA kits.

### Statistical Analysis

Shapiro–Wilk test was used for the normal distribution of the data and the homogeneity of group variances was examined by the Levene test. Mean and standard deviation were used as descriptors for normally distributed data. In independent group comparisons for 2 groups; independent samples *t*-test, and for more than 2 groups; when variances were homogeneous, one-way analysis of variance and Tukey pairwise comparison method, when variances were not homogeneous, Welch’s test and Tamhane’s T2 pairwise comparison method were used. Median, minimum, and maximum values were used in summarizing the score data. Mann–Whitney U-test, Kruskal–Wallis test, and Conover pairwise comparison method were used for independent group comparisons. The change in time was tested using the Wilcoxon paired 2-sample test. The significance level was accepted as 0.05 in all tests.

## Results

### Histological Findings

Within the scope of the experiment, SP group, UW group, and UW + Mel group were formed, liver tissues were removed from the subjects after sacrifice, and tissue samples were taken at 0 and 12^th^ hours.

In our study, a statistically significant difference was not observed within and between groups in terms of sinusidal dilatation, hydropic degeneration, and hepatocyte necrosis (Table 1), and there was no statistically significant difference between hours of the groups in terms of glycogen storage and PAS+ (Table 2).

While no statistically significant difference was observed between the 0 hour and the 12^th^ hour within the UW + Mel group, the 12^th^-hour immunoreactivity of the UW group was calculated to be significantly higher than the UW group 0 hour. When the UW group and UW + Mel group were compared at the 12^th^ hour, the Caspase 3 immunoreactivity of the UW group at the 12^th^ hour was found to be significantly higher (Table 2).

At 0 hour, the liver was evaluated in normal histological structure in sections stained with HE in all SP, UW, and UW + Mel groups. Poor Caspase 3 immunoreactivity was found in liver sections stained with Caspase 3 by the IHC staining method. In the sections stained with PAS, moderate and strong PAS+ staining was detected in the liver parenchyma.

**Figure 1.**
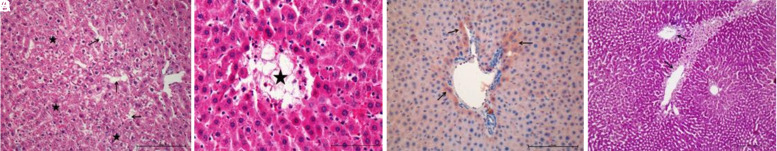
(A) The 12^th^-hour liver parenchyma of the UW group. Arrow, sinusoidal dilatation; asterisk: hydropic degeneration of hepatocyte cords (HE, 20×, size C = 100 µm). UW, University of Wisconsin. (B)The 12^th^-hour necrosis area of UW group. Asterisk, necrosis of hepatocytes (HE, 40×, size C = 50 µm). HE, hematoxylin and eosin; UW, University of Wisconsin. (C)The 12^th^-hour Caspase 3 reactivity of UW group. Arrow, strong Caspase 3+ staining in periportal hepatocytes (IHC, 20×, size C = 100 µm). IHC, immunohistochemical staining; UW, University of Wisconsin. (D)The 12^th^-hour PAS staining of UW group. Black arrow, weak PAS+ staining in pericentral first-line hepatocytes; white arrow, weak PAS+ staining in periportal first-line hepatocytes (PAS, 10×, size C = 100 µm). PAS, periodic acid Schiff; UW, University of Wisconsin.

At the 12^th^ hour of UW group, degeneration and sinusoidal dilatation were observed in hepatocyte cords in some sections stained with HE (Figure 1A). In this group, necrosis was detected in small hepatocyte groups in places (Figure 1B). The total histological damage score of this group was determined as 1 ± 0.8.

Moderate caspase 3+ staining was detected in IHC caspase 3 staining of this group. Strong caspase 3+ immunoreactivity was detected in pericentral and periportal hepatocytes (Figure 1C). The IHC Caspase 3 immunoreactivity score of this group was found to be 1.7 ± 0.5. In the sections stained with PAS, moderate and strong PAS+ staining was observed in the liver parenchyma similar to the other groups. Weak PAS+ staining was observed in some pericentral vein and first-line hepatocytes in the periportal area (Figure 1D).

**Figure 2.**
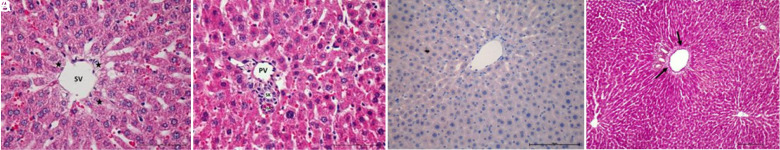
(A) UW + Mel group 12^th^ hour liver parenchyma. SV, central vein. Star, hydropic degeneration areas (HE, 20×, Size C = 100 µm). HE, hematoxylin and eosin; UW + Mel, University of Wisconsin + melatonin. (B)The 12^th^-hour portal area of UW+Mel group. Portal area in normal histological view. HA, hepatic artery; PV, portal vein; SK, bile duct; UW + Mel, University of Wisconsin + melatonin (HE, 40×, size C = 50 µm). (C)The 12^th^-hour Caspase 3 reactivity of UW + Mel group. Weak Caspase 3+ staining in the pericentral area (IHC, 20×, size C = 100 µm). IHC, immunohistochemical staining; UW + Mel, University of Wisconsin + melatonin. (D)The 12^th^-hour PAS staining of UW + Mel group. Arrow, poor PAS+ staining in periportal hepatocytes (PAS, 10×, size C = 100 µm). PAS, periodic acid Schiff; UW + Mel, University of Wisconsin + melatonin.

Although minimal hydropic changes and rarely minimal hepatocyte necrosis were observed in the pericentral first-line hepatocytes in the liver sections stained with HE at the 12^th^ hour of the UW + Mel Group, hepatocyte cords and sinusoid structures were generally observed in normal histological appearance (Figure 2A). The vein, artery, and bile duct structures in the portal triads were generally observed in normal histological appearance (Figure 2B). For the UW + Mel Group, the total histological damage score of the 12^th^-hour sections was determined as 0.5 ± 0.5.

Poor Caspase 3 immunoreactivity was found in liver sections of this group treated with IHC Caspase 3. Caspase 3 immunoreactivity positivity in pericentral hepatocytes was observed in a similar appearance to hepatocytes in other parenchymal areas in terms of staining intensity (Figure 2C). The IHC Caspase 3 immunoreactivity score of this group was determined as 1.2 ± 0.4. In the sections stained with PAS, similar to the other groups, moderate and strong PAS+ staining was observed in the liver parenchyma. However, weak PAS+ staining was observed in some first-line hepatocytes in the periportal area (Figure 2D).

### Biochemical Findings

Significant differences were observed when statistical evaluations were made between hours and between groups for MDA, GSH, TAC, and TOS levels in tissue. When we look at the 0-hour MDA levels, it was found to be significantly lower in the UW + Mel group among the groups. When the 12^th^-hour MDA levels were compared within the group, it was determined that they were statistically significantly different from each other. When the UW group and the UW + Mel group were compared at 0 and 12^th^ hours, a significant difference was found (Table 3).

When the 12^th^-hour GSH levels and TAC levels were compared, the UW + Mel group was found to be statistically significantly higher. When the 0-hour and 12^th^-hour GSH and TAC levels of the UW + Mel group were compared, a significant difference was found (Table 3).

Significant differences were also observed between TOS values. When the TOS levels were observed at 0 hour, the UW + Mel group was found to be statistically significantly lower. When TOS levels were compared at the 12^th^ hour, the UW group and UW + Mel group showed statistically significant differences. And when the 0-hour and 12^th^-hour TOS levels of the UW group were compared, a significant difference was observed (Table 3).

The 12^th^-hour serum liver function test (LFT) values were found to be statistically significantly lower in the melatonin group compared to the UW and control group (Table 4).

## Discussion

Preservation solutions that are used today cannot completely prevent cell damage, loss of function, and cell death after a certain period of time.^18^ For this reason, studies are continuing to increase the tissue protective properties of preservation solutions.

In preservation solutions, various antioxidants are added to the solution for tissue protection.^19,20^ However, considering that antioxidants increase cell resistance against oxidative stress, antioxidant supplementation to be applied to the donor before transplantation is likely to increase the resistance of cells in the transplanted organ against stress during transplantation. For this reason, in our study, we aimed to increase the resistance of the liver cells during the transplantation and during the adaptation process to the new organism and to protect their cellular integrity by supplementation of melatonin to donors at a certain time before the liver is taken from the donor.

The largest cell group of the liver parenchyma is hepatocytes, and preservation of the histological and biochemical properties of these cells is very important, especially in the success of liver transplantations.^21^ Various molecules that protect the properties of hepatocytes and substances with antioxidant properties are added to the preservation solutions used for this purpose. This study is considered to be different when compared with similar studies on this subject in the literature. In our study, melatonin, which is thought to benefit from the antioxidant effects of the liver in UW solution is given to the organism systemically before hepatectomy, instead of adding it to the preservation solution. In this way, by participating in the metabolism of the organism, melatonin increases the antioxidant capacity in the pathways in which it plays a role in cells before liver ischemia and enables it to perform an effective cytoprotective function. Considering the publications in the literature, it is seen that although melatonin varies according to the applied dose, it reaches its highest level in an average of 50 minutes after administration in the blood and its half-life is 45 minutes.^22^ Therefore, in our study, it was aimed to reach the highest level of melatonin in the blood tissue and thus in the liver tissue during hepatectomy by applying melatonin 45 minutes before hepatectomy.

There are very few studies on the subject with histological findings, and there is not enough histological, histochemical, and IHC data on the liver tissue during the preservation process in liver transplantation. However, in a study, histopathological changes such as necrosis, hepatocyte degeneration, sinusoidal dilatation, hemorrhage, vascular congestion, and dilatation in the liver tissue as a result of cold stress and an increase in oxidative stress parameters were found.^23^ In our study, hepatocytes and sinusoid structure in the liver tissue were evaluated histologically in detail in a 12-hour time duration in UW preservation solution and the findings were presented. The prominent findings were scored and compared between groups.

In a study, it was reported that melatonin protects glycogen storage in hepatocytes from the central vein toward the portal area and provides intense PAS+ staining.^24^ Studies show that melatonin maintains glycogen storage in liver tissue in any state of toxicity or oxidative stress.

At the 12^th^-hour PAS staining of the UW group and UW + Mel group, a moderate and strong PAS+ staining of the liver parenchyma was observed. At the 12^th^-hour of the UW group, PAS+ staining in the pericentral vein and first-line hepatocytes in the periportal area were observed to be at a weak level. We think that this is due to the absence of an ischemia process before hepatectomy in our experimental protocol, the fact that the rats were in normal and similar feeding conditions and that the liver tissues of all groups had similar post-hepatectomy ischemia and hypothermia conditions.

Apoptosis was defined as a significant indicator of ischemia-reperfusion-induced graft damage. A study showed that by inhibiting the apoptosis pathway, the normal function of hepatocytes and graft and cells is completely protected from apoptosis.^25^ These studies show that the prevention of apoptosis plays a critical role in the healthy maintenance of the liver graft in the recipient. Therefore, high Caspase 3 activity is a negative indicator of transplant success, while a low level positively affects the success of the transplant. Therefore, preventing apoptotic pathways that occur during the preservation process is of great importance in preventing graft rejection.

Literature data show that one of the main causes of failure in transplanted grafts is the increasing number of apoptotic hepatocytes with time.^26^ In our study, due to the increased apoptotic activity of hepatocytes in the UW group depending on the time, Caspase 3 immunoreactivity increased. However, in the melatonin group, Caspase 3 immunoreactivity was decreased by preventing hepatocytes from going to apoptosis. At this point, melatonin is applied before the organ transplantation and the presence of a higher amount of melatonin in the cell compared to normal cells and the cytoprotective activity of melatonin against cellular damage during the preservation process is thought to effectively protect the cell by suppressing the apoptosis pathway in the cell.

Serum LFT elevation, decreased bile production, and severe coagulopathy after liver transplantation are considered to be the defining factors for primer non-function and death is inevitable in all non-retransplanted cases.^27–29^ In a study, the protective properties of antioxidant supplements before transplantation during preservation were investigated. According to the findings given in this study, it is seen that LFT values are lower in the groups that were given prostaglandin E-1.^20^ Similarly, in our study, 12^th^-hour LFT values were found to be statistically significantly lower in the melatonin group compared to the UW group. When the findings are evaluated, it is thought that intraperitoneal melatonin administration before transplantation reduces cellular damage and provides the preservation of LFT values.

In their study, Hassan et al^30^ looked at the change in oxidative stress parameters in the transplanted liver after transplantation. This shows that as the transplanted graft completes the adaptation process, the MDA level, which is one of the damage parameters, decreases. When all the data obtained were evaluated, it was seen that melatonin administration before transplantation reduced the MDA level by preventing oxidative stress in the tissue during the preservation process.

In addition to the antioxidant function of GSH, it has regulating properties of signal transduction, cell proliferation, and immune response.^31,32^ In a study, GSH levels were measured in tissue samples during the preservation process and after transplantation. According to the findings, the GSH level at the end of cold preservation significantly decreased in the 2nd hour after transplantation.^33^ In our study, when the 12^th^-hour GSH levels were compared the UW + Mel group was statistically significantly higher. When the 0-hour and 12^th^-hour GSH levels of the UW + Mel group were compared, a significant difference was found.

One of the biochemical indicators of tissue damage is the level of TAC and TOS values. In cases such as I/R, TAC values decrease while SOR values increase. In the study conducted by Thorat et al^34^ it was stated that TAC decreased as a result of the increase in SOR in patients who underwent organ transplantation. Therefore, antioxidant supplementation to patients before transplantation may play an important role in reducing reperfusion damage. In our study, significant differences were observed in terms of TAC levels when the statistical evaluation was made within and between groups.

In a study in which the antioxidant activity of propofol and isoflurane was compared in liver transplant donors, preoperative, intraoperative, and postoperative plasma TOS levels were measured and plasma TOS levels increased in both groups in the intraoperative period according to preoperative values. However, in the postoperative period, the plasma TOS level decreased in the propofol group, while it remained almost the same in the isoflurane group compared to the preoperative values. In both groups, a decrease in postoperative TOS level was observed compared to intraoperative plasma.^35^ Significant differences were also observed between TOS values in our study.

## Conclusion

In UW solution, which is routinely used in liver transplantations, time-dependent cellular damage in the liver cannot be completely prevented. When all the findings obtained in our study were evaluated, it was determined that intraperitoneal melatonin administration before organ transplantation plays an important role in the IHC and biochemical protection of the liver tissue in preservation solution. However, by carrying out advanced multidisciplinary studies on the subject, it is necessary to clarify all aspects of the protective properties of melatonin administered intraperitoneally to the donor before transplantation.

## Figures and Tables

**Table 1.** Histological Findings of the Groups

		0^th^ Hour Median (Min-Max)	12^th^ Hour Median (Min-Max)	*P* (W)
Sinusoidal dilatation	SP	0 (0-0)		
	UW	0 (0-1)	0 (0-1)	.655
	UW + Mel	0 (0-1)	0 (0-1)	.157
	*P* (KW)	.407	*P* (M) = 1	
Hydropic degeneration	SP	0 (0-0)		
	UW	0 (0-1)	0 (0-2)	.257
	UW + Mel	0 (0-1)	0 (0-1)	1
	*P* (KW)	.407	*P* (M) = .121	
Hepatocyte necrosis	SP	0 (0-0)		
	UW	0 (0-0)	0 (0-1)	.157
	UW + Mel	0 (0-0)	0 (0-1)	.317
	*P* (KW)	1	*P* (M) = .542	
Total score	SP	0 (0-0)		
	UW	0 (0-1)	1 (0-2)	.058
	UW + Mel	0 (0-1)	1 (0-1)	.180
	*P* (KW)	.129	*P* (M) = .150	

KW, Kruskal–Wallis test; M, Mann–Whitney *U*-test; SP, serum physiologic; UW, University of Wisconsin; UW + Mel, University of Wisconsin + melatonin; W, Wilcoxon paired 2-sample test.

SP, n = 8; UW, n = 10; UW + Mel, n = 10.

**Table 2.** Caspase 3 and PAS Staining of All Groups

		0^th^ Hour Median (Min-Max)	12^th^ Hour Median (Min-Max)	*P* (W)
Caspase 3	SP	1 (1-1)		
	UW	1 (1-2)	2 (1-2)^a^	.025
	UW + Mel	1 (1-1)	1 (1-2)^b^	.157
	*P* (KW)	.154	*P* (M) = .005	
PAS	SP	2.5 (2-3)		
	UW	3 (2-3)	3 (2-3)	1
	UW + Mel	2.5 (2-3)	3 (2-3)	.739
	*P* (KW)	.883	*P* (M) = 1	

KW, Kruskal–Wallis test; M: Mann–Whitney *U*-test; PAS, periodic acid Schiff; SP, serum physiologic; UW, University of Wisconsin; UW + Mel, University of Wisconsin + melatonin; W, Wilcoxon paired 2-sample test.

SP, n = 8; UW, n = 10; UW + Mel, n = 10.

The superscripts show the difference between groups in each time period. Groups with different superscripts are statistically different.

**Table 3.** Oxidative Stress Parameters of All Groups

	Group	0^th^ Hour		12^th^ Hour		*P* (P)
		Mean	SD	Mean	SD	
MDA	SP	760.84^a^	25.74			
	UW	764.43^a^	14.59	635.51^a^	11.51	**<.001**
	UW + Mel	647.93^b^	6.46	564.46^b^	19.88	**<.001**
		P (ANOVA) < .001		*P* (I) < .001		
GSH	SP	950.77^a^	15.48			
	UW	952.24^a^	26.1	945.95^a^	10.01	.528
	UW + Mel	950.15^a^	29.5	1137.12^b^	38.89	**.001**
		P (ANOVA) = .988		*P* (I) < .001		
TAC	SP	3.53^a^	0.27			
	UW	3.66^a^	0.2	3.65^a^	0.37	.945
	UW + Mel	3.48^a^	0.31	7.01^b^	0.11	**<.001**
		P (ANOVA) = 0.500		*P* (I) < .001		
TOS	SP	47.75^a^	1.41			
	UW	47.64^a^	3.93	40.54^a^	5.07	**<.001**
	UW + Mel	39.78^b^	3.07	32.99^b^	0.97	**.007**
		P (ANOVA) < .001		*P* (I) = .014		

ANOVA, one-way analysis of variance; I, independent samples *t*-test; GSH, reduced glutathione; MDA, malondialdehyde; P, paired samples *t*-test; SD, standard deviation; SP, serum physiologic; TAC, total antioxidant capacity; TOS, total oxidant status; UW, University of Wisconsin; UW + Mel, University of Wisconsin + melatonin.

SP, n = 8; UW, n = 10; UW + Mel, n = 10.

The superscripts show the difference between groups in each time period. Groups with different superscripts are statistically different.*P* < .05 was accepted as significant.

**Table 4.** Serum AST, ALT, and ALP Parameters of All Groups

	Group	Mean	SD	*P* (ANOVA)
AST	SP	110.16^a^	6.29	**<.001**
	UW	96.10^b^	2.66	
	UW + Mel	79.79^c^	1.82	
ALT	SP	125.00^a^	2.83	**<.001**
	UW	115.33^b^	2.34	
	UW + Mel	96.33^c^	2.34	
ALP	SP	14.23^a^	1.09	**<.001**
	UW	11.82^b^	0.29	
	UW + Mel	8.71^c^	0.46	

ALP, alkaline phosphatase; ALT, alanine aminotransferase; ANOVA, one-way analysis of variance; AST, aspartate aminotransferase; SP, serum physiologic; UW, University of Wisconsin; UW + Mel, University of Wisconsin + melatonin.

SP, n = 8; UW, n = 10; UW + Mel, n = 10.

The difference between the groups with different superscripts is statistically significant. *P *< .05 was accepted as significant.
